# Dexamethasone-Loaded, PEGylated, Vertically Aligned, Multiwalled Carbon Nanotubes for Potential Ischemic Stroke Intervention

**DOI:** 10.3390/molecules23061406

**Published:** 2018-06-10

**Authors:** Patrick P. Komane, Pradeep Kumar, Thashree Marimuthu, Lisa C. du Toit, Pierre P. D. Kondiah, Yahya E. Choonara, Viness Pillay

**Affiliations:** 1 Wits Advanced Drug Delivery Platform Research Unit, Department of Pharmacy and Pharmacology, School of Therapeutic Sciences, Faculty of Health Sciences, University of the Witwatersrand, Johannesburg, 7 York Road, Parktown 2193, South Africa; patrickk@uj.ac.za (P.P.K.); pradeep.kumar@wits.ac.za (P.K.); thashree.marimuthu@wits.ac.za (T.M.); lisa.dutoit@wits.ac.za (L.C.d.T.); pierre.kondiah@wits.ac.za (P.P.D.K.); yahya.choonara@wits.ac.za (Y.E.C.); 2 Department of Applied Chemistry, University of Johannesburg, 27 Nind Street, Doornfontein, Johannesburg 2028, South Africa

**Keywords:** multiwalled carbon nanotubes, chemical functionalization, chemical vapor deposition, PEGylation, ischemic stroke article, yet reasonably common within the subject discipline

## Abstract

The complete synthesis, optimization, purification, functionalization and evaluation of vertically aligned multiwalled carbon nanotubes (VA-MWCNTs) was reported for potential application in dexamethasone delivery to the ischemic brain tissue. The conditions for high yield were optimized and carbon nanotubes functionalized and PEGylated prior to dexamethasone loading. Morphological changes were confirmed by SEM and TEM. Addition of functional groups to MWCNTs was demonstrated by FTIR. Thermal stability reduced following MWCNTs functionalization as demonstrated in TGA. The presence of carbon at 2θ of 25° and iron at 2θ of 45° in MWCNTs was illustrated by XRD. Polydispersive index and zeta potential were found to be 0.261 and −15.0 mV, respectively. Dexamethasone release increased by 55%, 65% and 95% in pH of 7.4, 6.5 and 5.5 respectively as evaluated by UV-VIS. The functionalized VA-MWCNTs were demonstrated to be less toxic in PC-12 cells in the concentration range from 20 to 20,000 µg/mL. These findings have demonstrated the potential of VA-MWCNTs in the enhancement of fast and prolonged release of dexamethasone which could lead to the effective treatment of ischemic stroke. More work is under way for targeting ischemic sites using atrial natriuretic peptide antibody in stroke rats.

## 1. Introduction

Carbon nanotube synthesis and their application in various fields have become more popular to date. Carbon nanotubes (CNTs) are a fairly new group of nanomaterials that have demonstrated promising results in various areas of nanomedicine due to their unique structures and outstanding physicochemical properties [1]. Based on their unique properties, CNTs have attracted much attention since a report by Iijima in 1991 [2]. CNTs are potential candidates for a variety of applications such as chemical sensors, nanoelectronics, catalyst support, targeting of diseased sites and drug delivery systems. The nature and quality of CNTs are highly dependent on the method of synthesis which regulates the graphitization degree, helicity and diameter [3].

A spectrum of procedures to produce CNTs have been reported, and the most commonly used procedures are arc discharge, laser ablation and chemical vapor deposition. The catalytic chemical vapor deposition (CCVD) is the most popular and preferred technique for the production of MWCNTs in large scale as it is inexpensive and can produce high quality MWCNTs [4]. Nebulized spray pyrolysis is an improved version of CVD method with the ultra-high frequency radiation used to nebulize reactants in solution to produce a reactant aerosol. The aerosol is carried into a high temperature furnace by a suitable carrier gas. The reaction is achieved by placing the reaction mixture in a reservoir with a piezo electric transducer at the base and then connected to a frequency generator [5]. CVD is divided into a floating catalyst method in which a catalyst is in a gas phase and a fixed catalyst method in which a catalyst is supported in a solid phase. In the floating catalyst method, there is no need for catalyst preparation as the catalyst particles are continuously formed [6]. A precursor, ferrocene, is dissolved in organic solvent which also serves as a carbon source. It is based on the hydrocarbon decomposition on metallic catalysts such as iron, nickel, cobalt and molybdenum [7].

Dexamethasone is a glucocorticoid with anti-inflammatory properties rendering it potentially effective for the treatment of inflammatory diseases including ischemic stroke. However, its therapeutic potential is negatively affected by the short circulatory half-life and poor pharmacokinetics requiring administration of higher doses which could lead to serious systemic side-effects [8]. In addition, dexamethasone could also cause hemorrhage and edema in stroke patients. If low doses of dexamethasone are conjugated to MWCNTs and targeted to the diseased site by a specific antibody, the current drawbacks may be overcome. Functionalization of these MWCNTs may increase biocompatibility, appendability of the drug to CNTs, and permeability of drug to cells and decrease the cytotoxicity of the CNTs.

In this study, the effect of various factors such as synthesis temperature, synthesis duration, gas flow rate, catalyst and location of silicon wafer in the furnace influencing the quality and yield of MWCNTs is investigated. For dexamethasone release studies, MWCNTs were first functionalized with acids, acylated, amidated and PEGylated prior to loading dexamethasone. The toxicity of the functionalized carbon nanotubes was evaluated using PC12 cells. Dexamethasone 21 phosphate was successfully loaded onto and released from the PEGylated VA-MWCNTs for the first time. These findings have demonstrated that VA-MWCNTs have a potentially important role in the fast and prolonged release of dexamethasone which could lead to an effective treatment of ischemic stroke. More work is under way for targeting ischemic sites using atrial natriuretic peptide antibody in stroke rats.

## 2. Results and Discussion

### 2.1. Location of the Substrate, Catalyst Concentration and Acid Purifiction of MWCNTs

**Figure 1:**
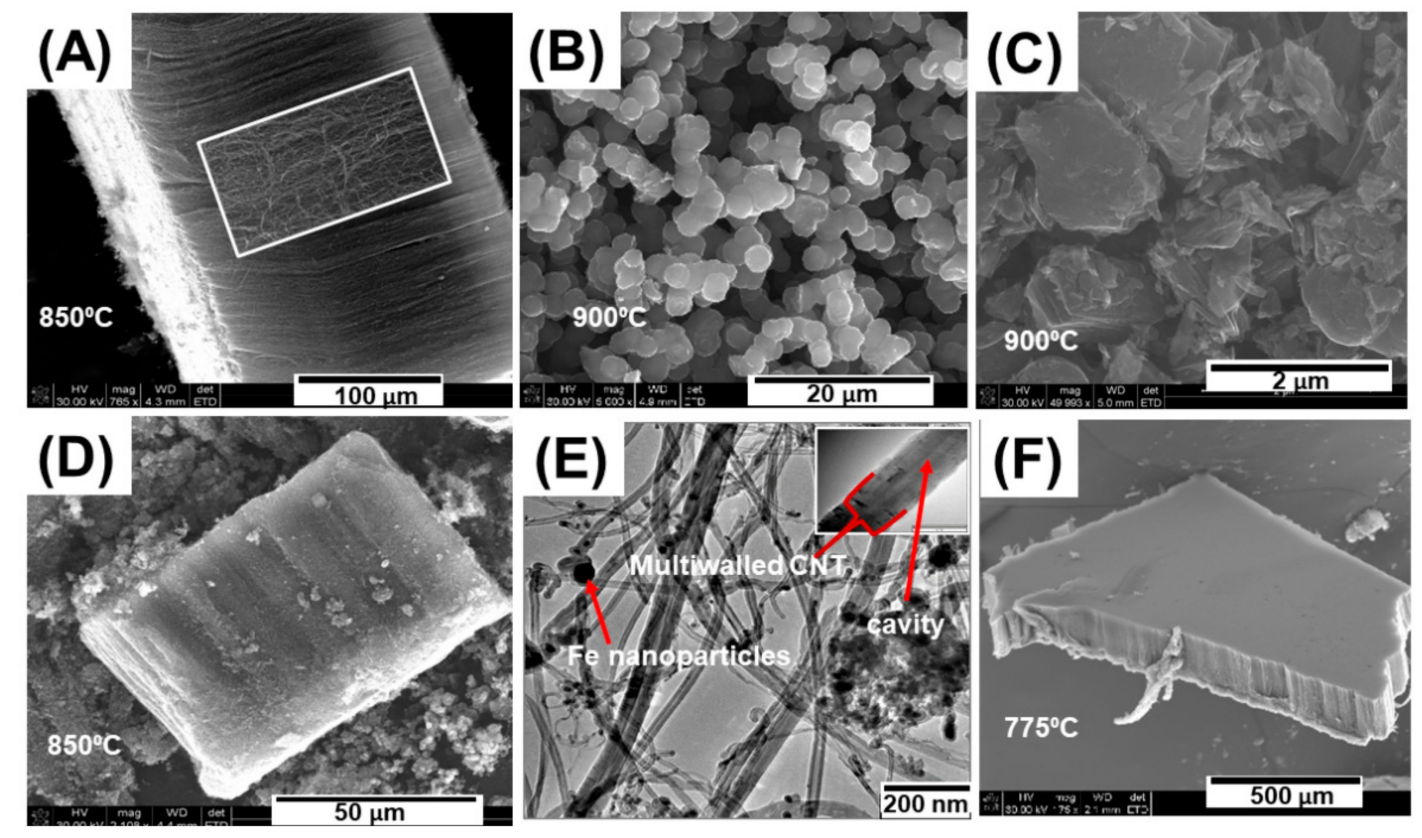
Vertically aligned multiwalled carbon nanotubes (MWCNTs) collected from silicon wafer 14 cm from the furnace inlet at 850 °C (**A**); Carbon nanospheres produced at 900°C (**B**); Carbon flakes from the furnace centre at 900 °C (**C**); MWCNTs from quartz tube walls at 850 °C (**D**); TEM micrograph of aligned MWCNTs (**E**) with magnified MWCNT insert. MWCNTs grown at 775 °C (**F**).

The location of the substrate, synthesis temperature, catalyst concentration, synthesis duration, argon flow rate were varied to determine the optimal pyrolysis conditions for the growth of MWCNTs. Aligned MWCNTs were successfully grown on the silicon wafer when it was placed 14 cm from the furnace inlet (Figure 1A,D,F). Carbon nanospheres and graphitic flakes were obtained when Si substrates were placed at the furnace centre (Figure 1B,C). MWCNTs consisting of more than one wall and iron nanoparticles were observed in their cavities (Figure 1E) and well aligned MWCNTs were obtained when temperatures of 850 °C and 775 °C were used (Figure 1A,F) whereas higher temperatures around 900 °C led to the formation of carbon flakes and nanospheres (Figure 1B,C).

Temperatures of 775 °C and 850 °C were found to be the optimal temperatures for iron atom mobility to form catalytic particles and toluene decomposition to form carbon and nucleate MWCNTs. At temperatures below 750 °C, the iron atoms were not mobile enough to aggregate together into particles to nucleate and grow nanotubes. At temperatures above 850 °C, the catalyst becomes highly mobile and quickly agglomerates into metal particles that are too large to initiate carbon nanotube nucleation, resulting in a process called ‘carbon overcoating’ [9] which results in the formation of carbon nanospheres and flake-like structures (Figure 1B,C).

**Figure 2:**
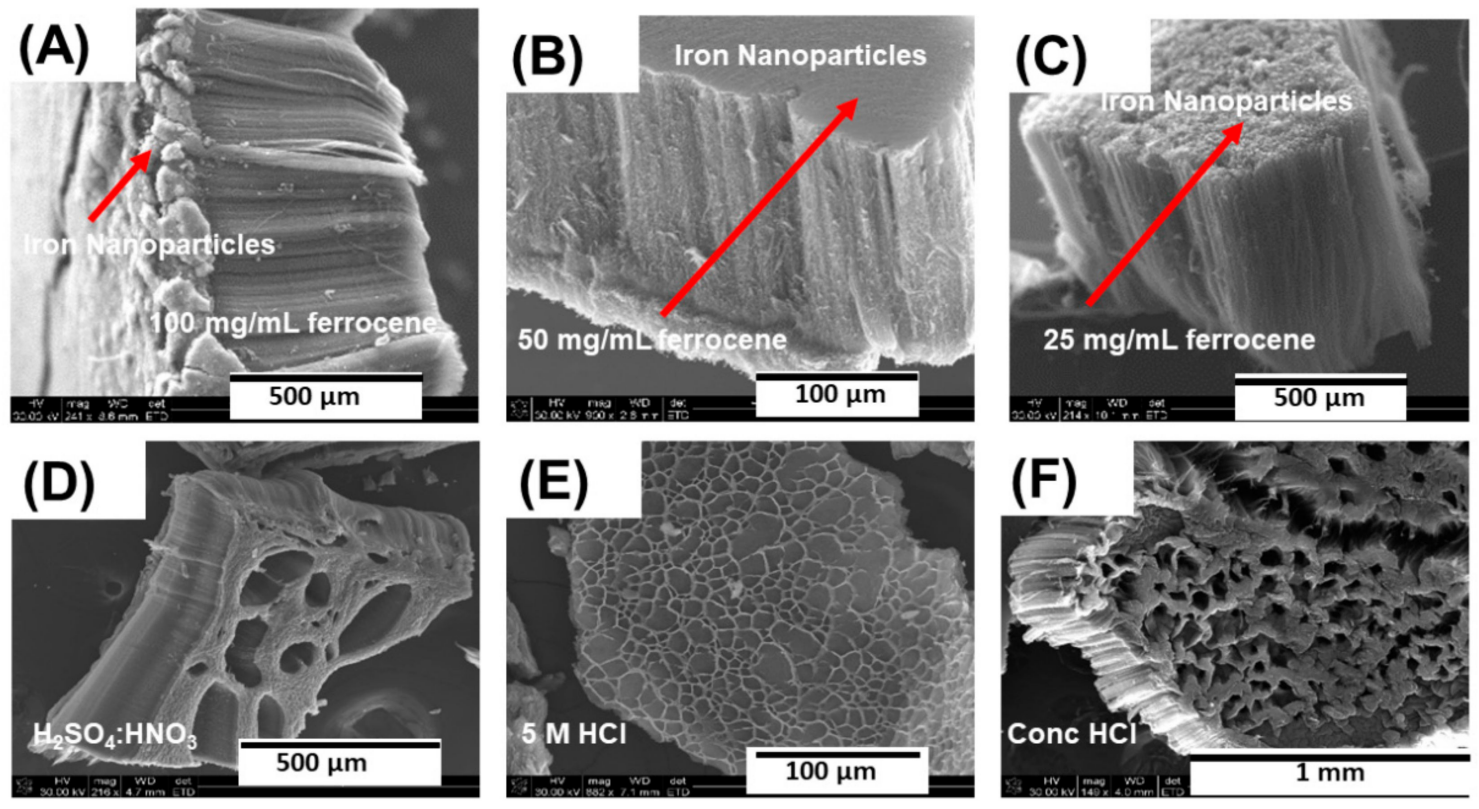
MWCNTs at various catalyst concentrations and purified with different acids. MWCNTs synthesised in 100 mg/mL ferrocene (**A**); MWCNTs prepared in 50 mg/mL ferrocene (**B**); MWCNTs when 25 mg/mL ferrocene was used (**C**). MWCNTs treated with different acids namely concentrated H_2_SO_4_:HNO_3_ (3:1) (**D**); 5 M HCl (**E**) and concentrated HCl (**F**).

Composition and concentration of the catalyst are important in MWCNT synthesis. A lower concentration leads to SWCNT formation, while a higher concentration leads to MWNT formation [10]. Different concentrations of ferrocene in toluene (25, 50 and 100 mg/mL) was used to evaluate the effect of the content of transition metal on the growth of MWCNTs. The thickness of MWCNTs increased with an increase in the ferrocene content. When 25 mg/mL of catalyst was used, MWCNTs with clean surfaces were produced with less or no iron nanoparticle layers on their surfaces (Figure 2A–C).

Ghaharpour and colleagues have demonstrated in their work that the higher content of catalysts provided better conditions for the growth of carbon nanotubes [11]. In this study, a higher concentration of catalyst (100 mg/mL) produced a thick layer of iron nanoparticles on the surface of MWCNTs (Figure 2A), whereas a thin layer was noticed when 50 mg/mL ferrocene was used (Figure 2B). There was no formation of iron nanoparticle layer when 25 mg/mL ferrocene was used as a catalyst (Figure 2C). Furthermore, acid purification eliminated the contaminants such as amorphous carbon and iron nanoparticles from carbon nanotubes.

VA-MWCNTs were treated with different acids for purification to remove amorphous carbon and residual iron. In addition, the treatment was performed to increase the dispersibility and hydrophilicity of the MWCNTs. When MWCNTs were subjected to HNO_3_:H_2_SO_4_ (1:3), holes with mean diameter of 104.31 µm were formed on MWCNTs (Figure 2D). A mixture of HNO_3_ and H_2_SO_4_ oxidized the iron nanoparticle, amorphous carbon and MWCNTs, leading to the formation of holes on MWCNTs. When MWCNTs were exposed to 5 M HCl, honeycomb-like structures with a mean diameter of 12.30 µm were generated (Figure 2E). Concentrated HCl resulted in the oxidation of MWCNTs leading to the formation of holes with mean diameter of 85.82 µm and shrinking on the surface of MWCNTs (Figure 2F).

### 2.2. Determination of Crystallinity, Thermal Properties and Raman Spectroscopy of MWCNTs

**Figure 3:**
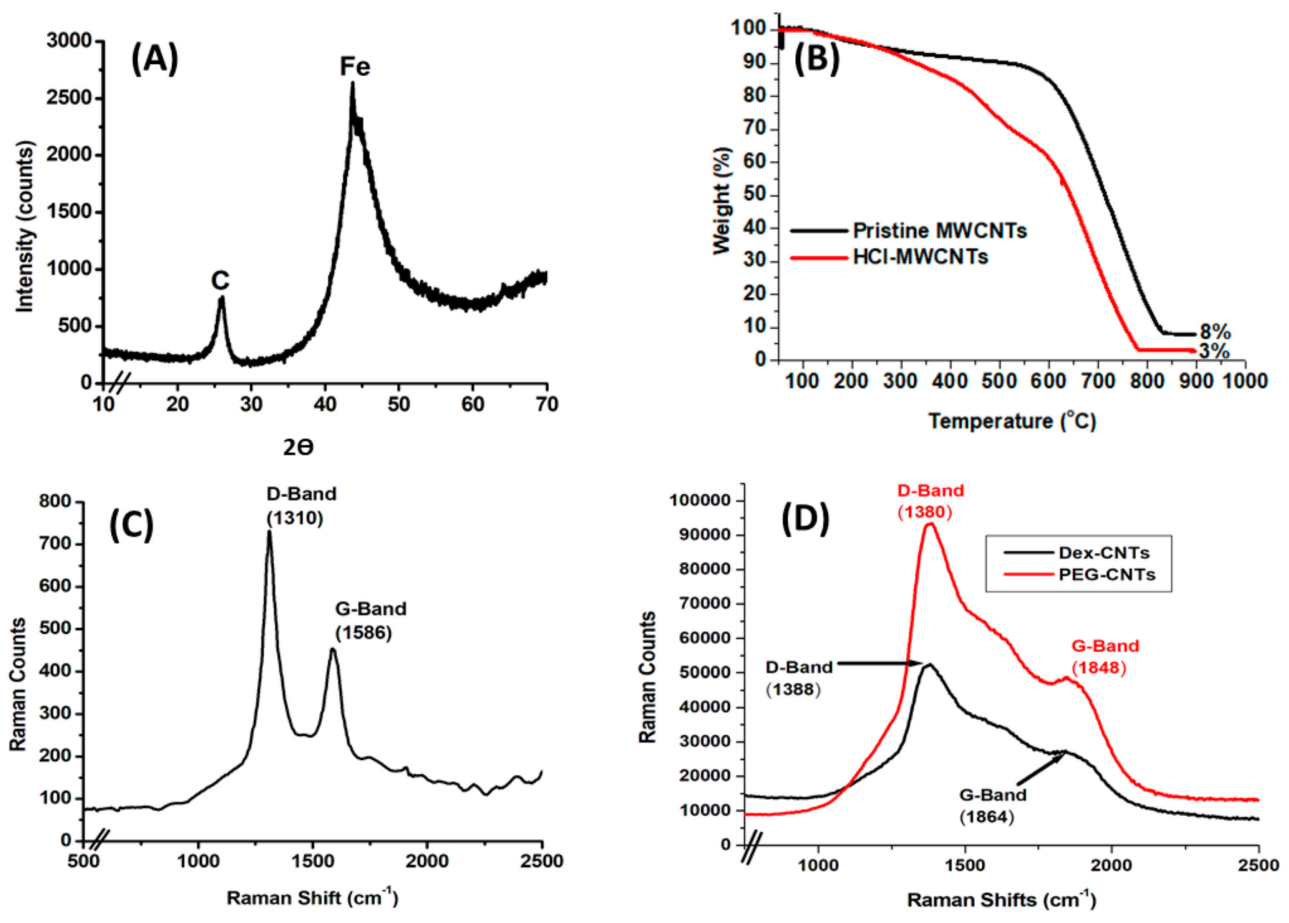
XRD diffraction pattern, TGA and Raman spectra of MWCNTs. Diffraction pattern of pristine MWCNTs (**A**); TGA of both pristine and HCl-treated MWCNTs (**B**); Raman spectrum of pristine MWCNTs (**C**) and Dex and PEG-CNTs (**D**).

Two characteristic peaks were observed at the angle (2θ) of 25° and 45°, confirming the presence of carbon in MWCNTs and iron in ferrocene, respectively (Figure 3A).

Thermal stability and purity of MWCNTs were determined using a thermogravimetric analyser. While the TGA curve of the pristine MWNTs showed no significant mass loss up to 600 °C, one sharp mass loss for the functionalized samples was observed in the temperature range from 150 to 775 °C. The sharp mass loss observed in the temperature range of the functionalized samples could be attributed to the HCl on the MWNTs surface, unreacted carboxylic acid groups as well as MWNTs that were oxidized in carboxylation stage (Figure 3B).

Acid functionalized MWNTs showed the remaining weight to be around 3% at 775 °C, which is attributed to the metal catalyst or the ash of MWNTs. Pristine MWNTs showed the remaining weight to be ~8% at 825 °C. Significant weight loss started at 150 °C and 600 °C in acid functionalized and pristine MWCNTs, respectively (Figure 3B). Complete decomposition occurred at 725 °C and 825 °C in acid functionalized and pristine MWCNTs, respectively. The findings confirm a successful purification of MWCNTs as depicted by fewer remains in the functionalized MWCNTs as opposed to impure pristine MWCNTs.

MWCNTs were PEGylated and their Raman spectra determined by Raman Micro200 (Perkin Elmer, Waltham, MA, USA). The D and G bands were observed in the non-PEGylated MWCNTs at 1310 cm^−1^ and 1586 cm^−1^, respectively (Figure 3C). A shift of both the D and G bands to the right was observed when MWCNTs were functionalized with PEG and dexamethasone as compared to the non-PEGylated CNTs (Figure 3D). There was a significant rise in the D band intensity and a drop in the G band intensity following functionalization. Our data agree with the work done by Voge and colleagues as they also observed a shift of D and G bands to the right in the functionalized MWCNTs [12]. Loading of dexamethasone caused a drastic decrease in the intensities of both bands and this could be due to the linkage that occurred between the PEGylated CNTs and dexamethasone.

### 2.3. Effect of Synthesis Times and Gas Flow Rates on the Length and Diameter of MWCNTs

**Figure 4:**
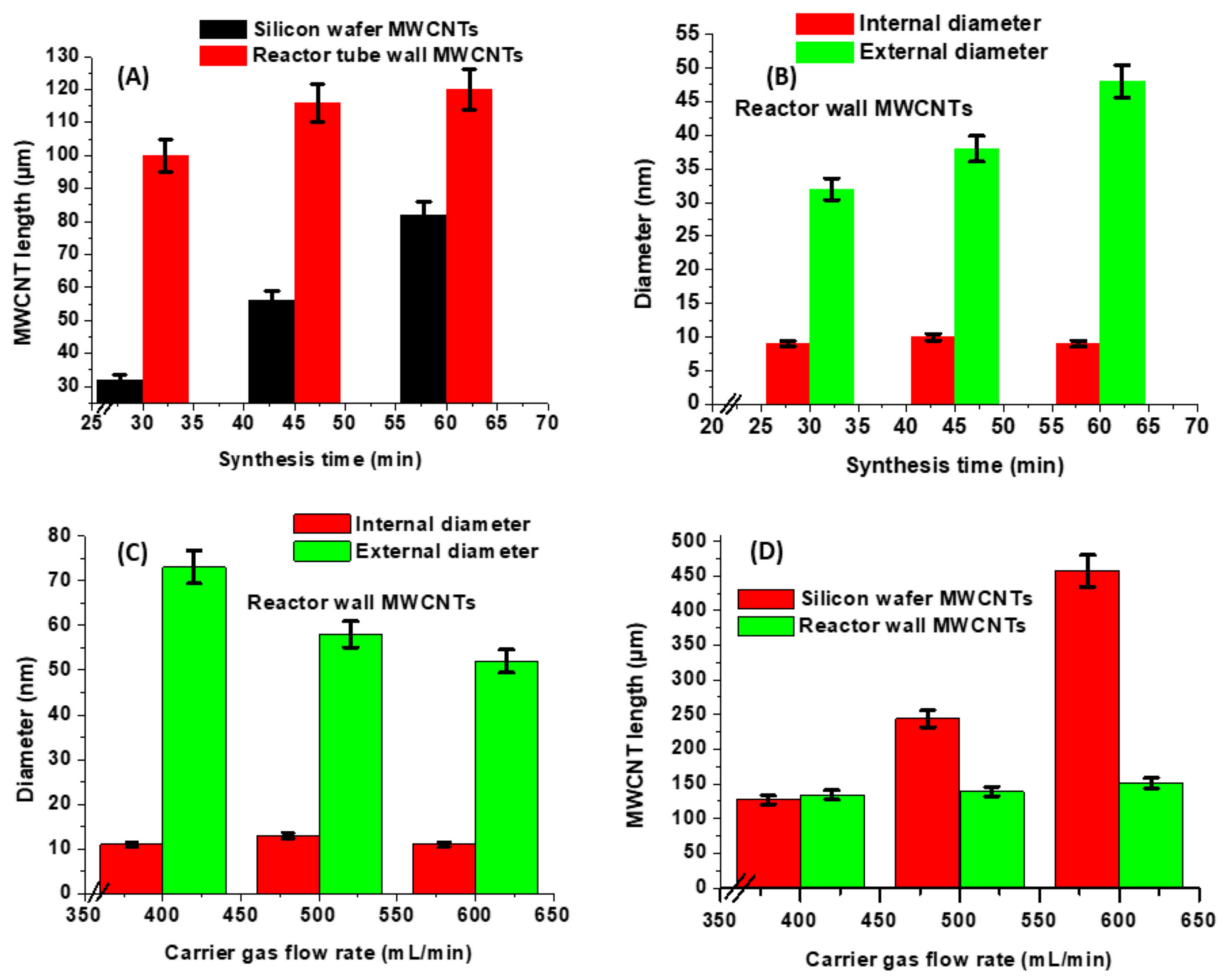
Synthesis parameters of MWCNTs. Synthesis time on the length or thickness of MWCNTs on both the silicon wafer (black column) and the reactor tube walls (red column) (**A**); synthesis time on both the MWCNTs internal (red column) and external (green column) diameters (**B**); carrier gas flow rate on both the MWCNTs internal (red column) and external (green column) diameters (**C**); carrier gas flow rate on MWCNTs length on both silicon wafer (red column) and reactor wall (green column) (**D**).

When different reaction times were employed in the synthesis of MWCNTs, a linear relationship was noticed between reaction time, length and external diameter of carbon nanotubes as demonstrated in Figure 4A,B. An increased catalyst annealing duration led to higher concentration of iron nanodroplets which enabled faster diffusion of carbon leading to more vertically aligned carbon nanotubes and higher growth efficiency. Rahman and colleagues synthesized aligned SWCNTs using annealing times of 10 s, 3 min, 10 min and 15 min. They found that the height of the SWCNT array increased from 229 µm to 246.5 µm as the catalyst annealing time was increased from 3 min to 10 min [7].

In this study, various flow rates of argon ranging from 400 to 600 mL/min were used and a linear relationship (R^2^ = 0.99711) between the gas flow rate and thickness was observed (Figure 4D). The carrier gas flow rate influenced the distribution of the carbonaceous products on the quartz tube walls and substrate. Tewari and Sharma investigated the effect of carrier gas flow rates of argon, nitrogen and ammonia on the yield of carbon nanotubes and found that there was a direct relationship between the flow rate and the yield of carbon nanotubes [6]. In this study, 95% argon balanced with 5% hydrogen was used to produce quality carbon nanotubes. There was no effect on the internal diameter of the MWCNTs as the synthesis time and gas flow rate were increased (Figure 4B,C). In this work, iron nanoparticles of 10 nm in diameter was used and the MWCNTs produced were of the internal diameter of around 10 nm. The internal diameter of MWCNTs remained constant as it was restricted by the size of iron nanoparticles.

As the synthesis time increased, the walls on the MWCNTs increased in number leading to an increase in external diameter (Figure 4B) as the external diameter is entirely dependent on the growth period of the carbon nanotubes. The external diameter decreased with an increase in the carrier gas flow rate as a result of carrier gas molecules occupying the catalyst sites to a greater degree than toluene molecules. Khorrami and Lotfi reported that an increase in the carrier gas flow rates decreased the formation of MWCNTs, because carrier gas molecules occupied catalyst sites more than ethanol molecules leading to the reduction of external diameter of the MWCNTs [13]. In this study, the external diameter dropped with a rise in gas flow rate as demonstrated in Figure 4C.

### 2.4. Mixture of Ferrocene/Nickelocene/Cobaltocene on MWCNT Growth

Bimetallic nanoparticles are more reliable catalysts than single element catalysts because both metals in the bimetallic nanoparticles can enhance certain functions by playing complementary catalytic roles [14]. The yield of CNTs increases many folds in comparison to using monometallic catalysts. It has been found that, whilst all the metals are able to produce CNTs, iron catalysts gave a large yield. The superior performance of the iron catalyst was attributed to iron higher carbon solubility, which helps to promote the production of CNTs [15]. In this study, a combination of ferrocene and cobaltocene resulted in higher total yield of MWCNTs than ferrocene alone and a mixture of both ferrocene and nickelocene. When a trimetallic catalyst was used, there was a slight reduction in the yield but still higher than ferrocene alone (Table 1).

### 2.5. Porositometric and Particle Size Distribution Properties of MWCNTs

**Figure 5:**
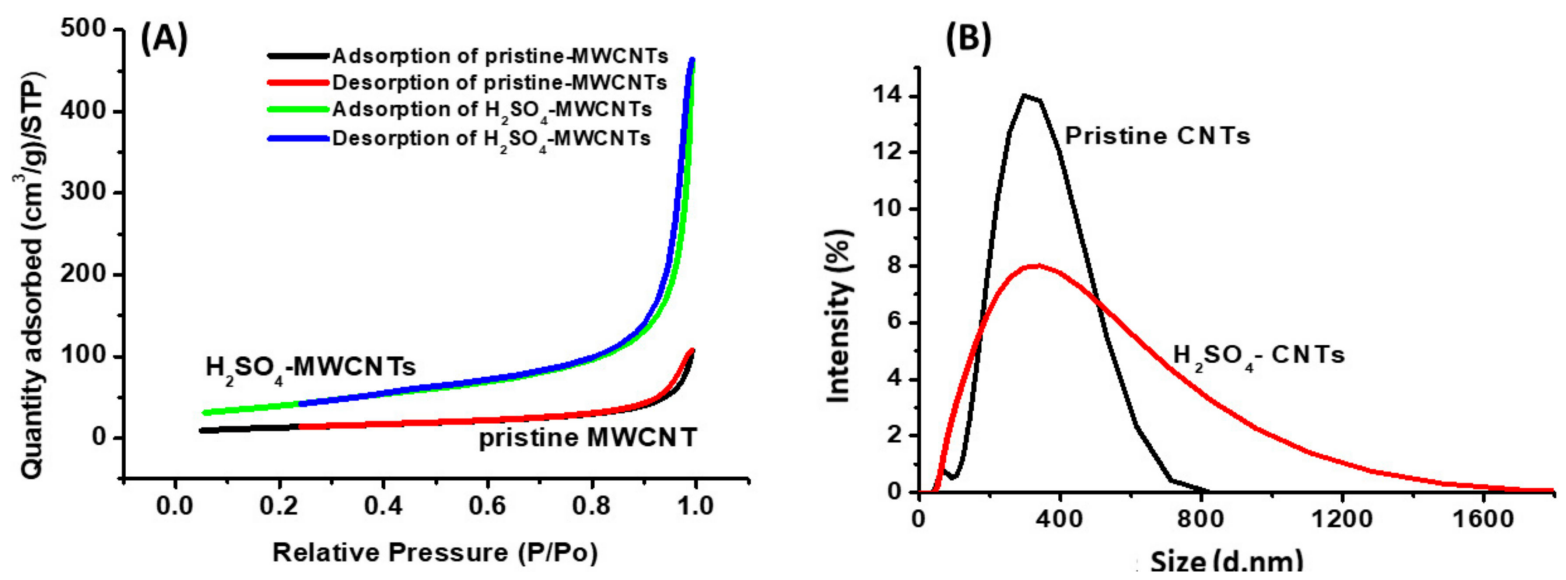
Porositometric and particle size properties of MWCNTs. BET isotherms for both the pristine (black curve for N_2_ adsorption and red curve for desorption) and HCl-treated (green curve for N_2_ adsorption and blue curve for desorption) MWCNTs (**A**). Particle size distribution of pristine and H_2_SO_4_-treated (for improving dispersibility and hydrophilicity) carbon nanotubes (**B**).

Following functionalization of CNTs with H_2_SO_4_, surface characteristics of both the pristine and functionalized MWCNTs were determined by a surface area and porosity analyser. BET measurements of MWCNTs are summarized in Table 2 and isotherms are demonstrated in Figure 5A. These isotherm plots display the Type IV adsorption as there is formation of monolayers by capillary condensation on mesoporous surfaces of the CNTs. The black and green curves indicate the adsorption of liquid nitrogen and the red and blue curves indicate desorption of liquid nitrogen from the CNTs. The pristine and H_2_SO_4_ functionalized MWCNTs were found to be mesoporous according to IUPAC based on their pore size range of 13 to 18 nm.

Acid treatment improved MWCNTs surface area as demonstrated by Figure 5A. Acid treatment increased the surface area, pore size and pore volume of the MWCNTs by 67.27%, 27.64% and 76.32%, respectively as depicted in Table 2.

CNTs are hydrophobic in nature and their dispersibility is achieved by functionalization by attachment of polar groups such as carbonyl (–C(O)–), carboxyl (–COOH), and hydroxyl (–OH) [16]. 

The data obtained confirmed the hydrophobic nature of the pristine MWCNTs and hydrophilic nature of the H_2_SO_4_ functionalized MWCNTs.

A Zetananosizer ZS (Malvern Instruments, Worcestershire, UK) was used to determine particle size distribution and zeta potential of MWCNTs. Zhao and colleagues demonstrated that defects in MWCNTs were more likely to form and functional groups were successfully grafted onto MWCNTs after oxidation. They also showed that the functionalized MWCNTs with negative groups as observed in Zeta potential measurements had superior suspension stability and could remain as a colloidal solution for 30 days [17]. In this study, the H_2_SO_4_ functionalized MWCNTs were suspended in deionized water at a concentration of 0.2 mg/mL and particle size distributions and zeta potentials were measured. The Z-average diameter and polydispersity index (PDI) were 287.08 nm and 0.261, respectively. This PDI indicates that the MWCNTs have a moderate size distribution. The zeta potential and conductivity were −15.0 mV and 0.0213 mS·cm^−1^ respectively. The MWCNTs possess negative charge on their surface and the zeta potential magnitude of 15 implies that the MWCNTs are slightly agglomerated.

Nanoparticles possess properties that depend entirely on their dimensions. There is currently a plethora of methods for surface characterization of nanoparticles. The PSD values of the MWCNTs are different from SEM and TEM values as the techniques operate differently to measure the dimensions of the particles (Figure 5B). For particle size distribution (DLS), the particles are suspended in a solution and the measurements are performed while the particles are in solution based on Brownian motion. The individual particles may agglomerate leading to an increase in the hydrodynamic diameter.

In SEM, the particle is in a solid state (dry condition) and the length of the individual particle could be determined easily. For TEM, the particle is also in a solid state and the length of the particle could be determined depending on the size (if too long, this could be determined by SEM). In TEM, the internal and external diameters could be determined as this technique has a better resolution compared to SEM. In general, SEM is suitable for large particles (above 50 nm in diameter) while TEM offers accurate results in small particles. DLS is a suitable technique for particles in solutions but inappropriate for polydispersive and heterogenous samples [18].

### 2.6. FTIR Spectroscopy and H^1^ NMR Analyses of PEGylated MWCNTs

**Figure 6:**
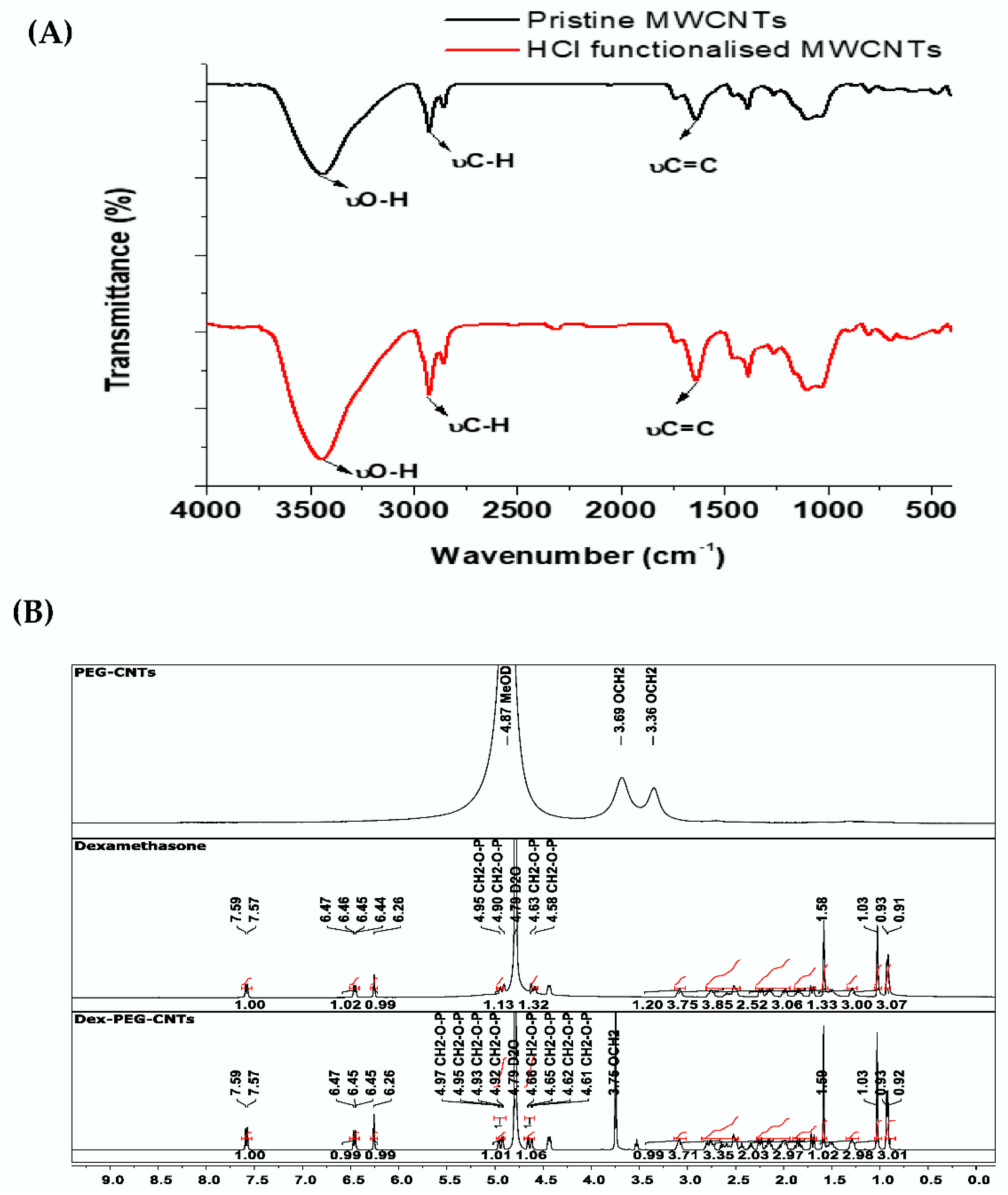
Molecular spectral properties of pristine and functionalized MWCNTs. FTIR spectra of pristine (red spectrum) and HCl functionalised (black spectrum) MWCNTs (**A**); ^1^H-NMR spectra of PEG-CNTs, Dexamethasone, and Dex-PEG-CNTs (**B**).

Relative to the spectrum for pristine MWCNTs, Figure 6A, the intensity of O-H and C-H stretching bands increased and broadened in the spectrum of MWCNTs-HCl, Figure 6A, due to acid treatment peaks that were observed at 1638 cm^−1^ which could be ascribed to C=C, respectively, in both the pristine and functionalized MWCNTs. [7].

In the ^1^H-NMR spectrum of PEG-CNTS (Figure 6B) there are chemical shifts observed only at δ 3.69 and 3.36 ppm which can be ascribed to the methylene protons on the PEG backbone [19]. The other proton signals were too weak and therefore not observed due to limited solubility of the PEG-CNTS in methanol. Relative to the spectrum of PEG-CNTS (Figure 6B) there are several new signals in the spectrum of Dexamethsone-21-phosphate loaded-PEG CNTS (Figure 6B) due to the presence of the loaded drug. For example, the presence of downfield protons 6.26 ppm (C=C-H) and 6.43 ppm are indicative of the steroid ring A system.

**Scheme 1:**
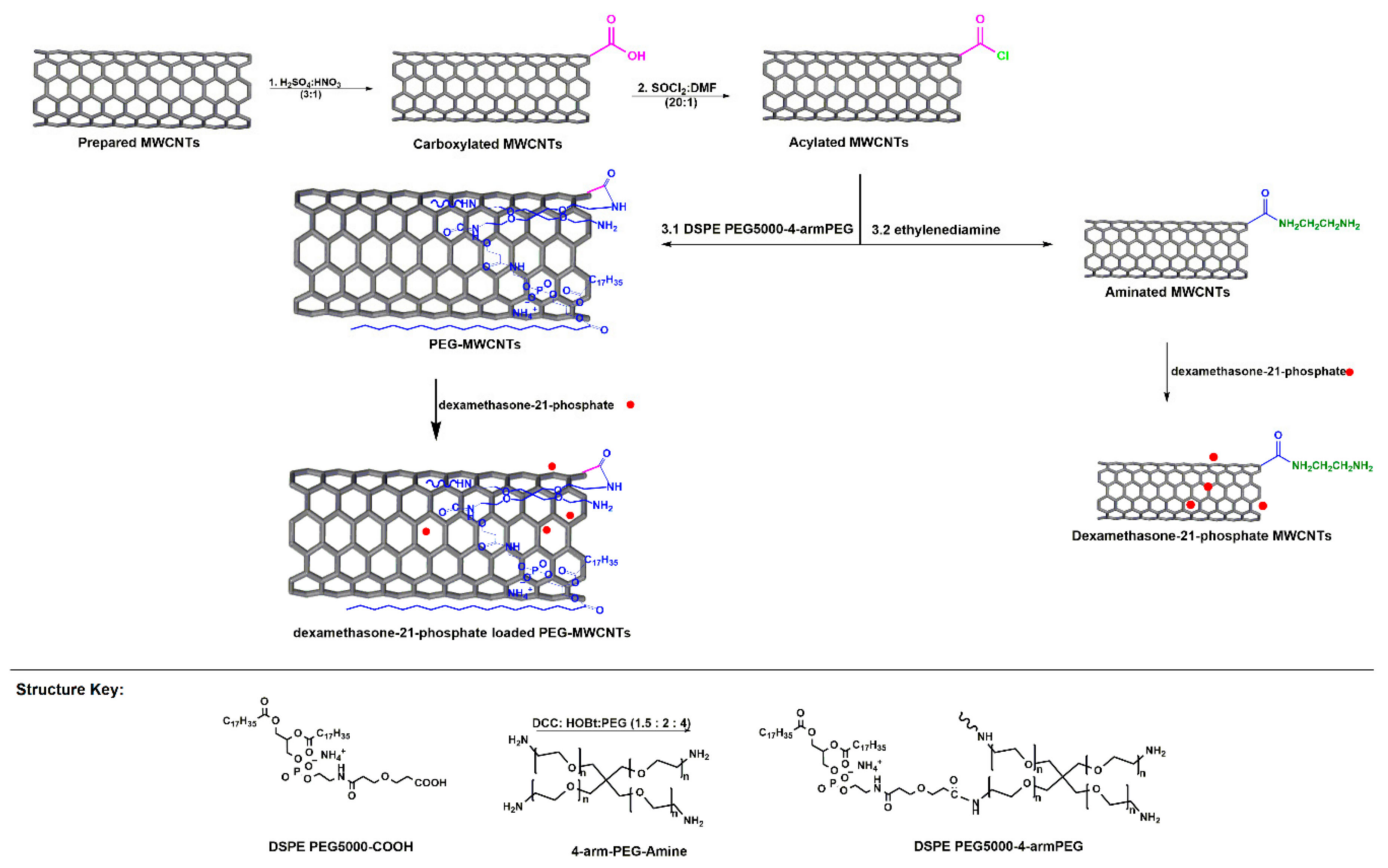
Schematic illustration showing the preparation of PEG-MWCNTs.

Relative to the spectrum of Dexamethsone-21-phosphate (Figure 6B) there are similar chemical shifts observed in the spectrum of Dexamethasone-21-phosphate loaded-PEG CNTS (Figure 6B). Furthermore, these signals show no significant shifts. Chemical shift at 3.75 ppm represents methylene protons on the PEG backbone (Figure 6B) and confirm the presence of the CNTs. Therefore, based on the ^1^H-NMR observations it can be proposed that the drug has being loaded into the CNT carrier (Scheme 1). The proposed loading sites and mechanism of loading that the drug is present in the formulation, where it may reside on the surface, inside the cavity of the tube or possible covalent interactions may occur as there are some (4.94, 4.64 ppm) slight upfield shifts (4.93, 4.61 ppm) [20].

### 2.7. Dexamethasone Loading to and Release from MWCNTs

Pristine MWCNTs are limited in drug delivery systems due to their poor drug entrapment capability [21]. The successful entrapment of dexamethasone was reported which is enabled due to the novel functionalization carried out. PEG-MWCNTs and non-PEG-MWCNTs were tested using dexamethasone-21-disodium phosphate because of the increased solubility of the phosphate derivative.

**Figure 7:**
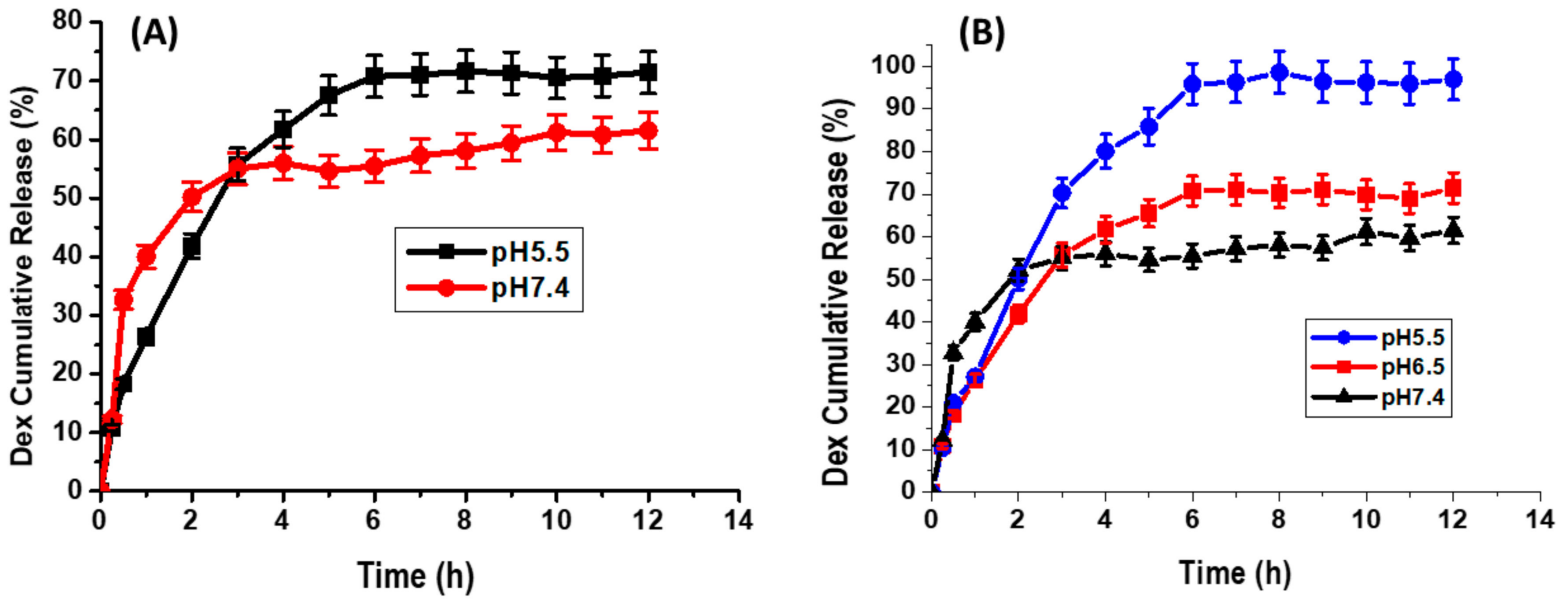
Dexamethasone release curves. Dex-MWCNTs in PBS buffer with pH 7.4 and pH 5.5 (**A**); Dex-PEG-MWCNTs in PBS buffer with pH 7.4, pH 6.5 and pH 5.5 (**B**).

Dialysis tubing over 12 h at various time intervals indicated the initial release of dexamethasone from MWCNTs after 0.5 h in PBS buffer with pH 5.5 and pH 7.4 in non-PEGylated MWCNTs. Maximum dexamethasone release occurred after 2 h and 6 h following dialysis in an basic and acidic milieu, respectively, and remained constant up to 12 h in PEGylated MWCNTs (Figure 7A). A change in the release pattern of dexamethasone from MWCNTs was observed in the PEGylated MWCNTs (Figure 7B). An initial gradual release of dexamethasone occurred after 0.5 h following dialysis in all PBS buffers. Maximum dexamethasone release was observed after 2 h in pH 7.4 PBS buffer 6 h in both pH 6.5 and 5.5 and remained constant thereafter. Only 55% of dexamethasone loaded on non-PEGylated MWCNTs were released in PBS buffer with pH 7.4 and 70% released in a buffer with pH of 5.5 (Figure 7A). Venditti and colleagues, in their work, loaded dexamethasone on gold nanoparticles and only 70% of the loaded drug was released in 5 days [22]. Roozbahani and colleagues studied dexamethasone release from laponite nanoplates. In their work, 47% of dexamethasone was released at pH = 7.4 while 76% was released at pH = 5.4 after 3 days [23].

In our study, a fast release with more than 50% was observed after 4 h. Drug remained entrapped due to weak interaction in CNT cavities and cannot achieve 100% release as a result.

Dexamethasone release was notably improved in PEGylated MWCNTs with 55%, 65%, 95% release in PBS buffers with pH 7.4, 6.5 and 5.5, respectively (Figure 7B), as compared to non-PEGylated MWCNT. It is possible that the slow release observed in Figure 7B is due to the ionic interaction between the opened charged MWCNTs and dexamethasone [24].

There are two possible mechanisms that could have taken place: namely drug loading as a result of entrapment into the inner cavities of the carbon nanotubes and ionic interaction as a result of phosphate derivative. The mechanisms of dexamethasone release from PEGylated MWCNTs in acidic and basic milieu have been demonstrated to be different.

The burst and subsequent sustained release could play a vital role in the therapeutic treatments, as the initial fast release could rapidly afford a therapeutic dose, and the succeeding sustained release could preserve the therapeutic dose for a prolonged period. The above findings have demonstrated an improved sustained release of dexamethasone when loaded to the PEGylated MWCNTs.

### 2.8. Cytotoxicity Evaluation of the Functionalized Carbon Nanotubes

Pristine carbon nanotubes are toxic, but their toxicities are reduced when they undergo functionalization. PC-12 cell lines were successfully grown in RPMI 1640 medium (Figure 7) and incubated with various concentrations of dexamethasone and functionalized carbon nanotubes for 24 h.

Zeinabad and colleagues studied the effects of SWCNTs and MWCNTs in PC-12 cells using concentrations ranging from 0.01 to 100 µg/mL. SWCNTs induced higher in vitro cytotoxicity against PC-12 as compared to MWCNTs as demonstrated by MTT assay. A decline in cell viability was observed as the concentration of the carbon nanotubes was increased [25].

Gopinathan and colleagues developed poly(ε-caprolactone)-based nanocomposite (PCL) scaffolds and evaluated their toxicity in PC-12 cells using a Cytox 96 cytotoxicity assay kit. Cell viability analyses demonstrated that there was no cell death within 48 h of incubation of PC-12 cells with the PCL nanocomposites [26]. In our study, PC-12 cells were seeded in 96 well plate at a concentration of 6 × 10^4^ cells/mL/well and incubated with various concentrations of dexamethasone and functionalized carbon nanotubes ranging from 20 to 20,000 µg/mL.

**Figure 8:**
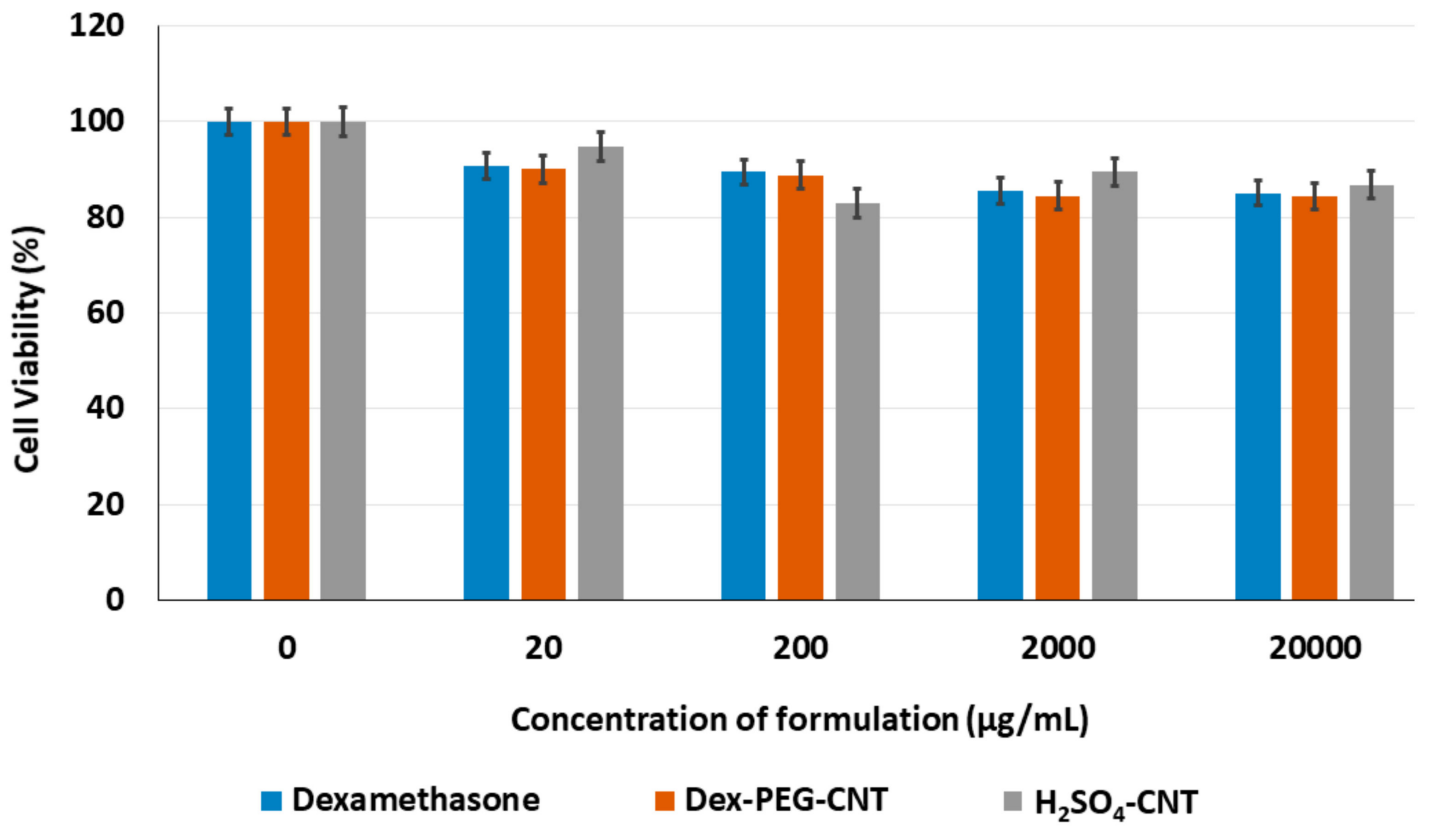
Cell viability following incubation with various concentrations of functionalized carbon nanotubes. H_2_SO_4_-MWCNTs were used as they are highly dispersible and hydrophilic. Blue column = free dexamethasone, brown column = equivalent dexamethasone concentration coupled to PEGylated MWCNTs and grey column for sulfonitric acid functionalized MWCNTs without dexamethasone. Concentrations of each formulation expressed as µg/mL from 0 to 20,000.

The average diameter of the cells was found to be approximately 50 µm as depicted in Figure 8. Cell viability was reduced from 100% to approximately 90% as demonstrated in Figure 8. The 10% decrease in viability demonstrated the very low cytotoxic effect of the functionalized carbon nanotubes. Therefore, these functionalized carbon nanotubes could be utilized as nanocarriers of anti-inflammatory drugs such as dexamethasone at a very low dose to the ischemic site in the stroke patient leading to the effective treatment of stroke.

## 3. Materials and Methods

### 3.1. Synthesis of MWCNTs Using Chemical Vapor Deposition

A circular silicon wafer (single side polished, <100>, N-type, contains No dopant, diam. × thickness 2 in. × 0.5 mm) from Sigma-Aldrich Corporation (St Louis, MO, USA) was dissected into 1 cm × 1 cm pieces and sonicated in acetone, methanol and water for 5 min. The silicon wafer was loaded into the quartz reactor tube to act as a platform for MWCNTs growth. The quartz tube was then inserted in a horizontal high temperature tube furnace (Elite TSH 12/38/500-2216E, Leicestershire, UK). The reaction vessel was activated with 40 mL catalyst and 95% baseline argon balanced with 5% hydrogen was used as a carrier gas. Flow rate was set to 50 mL/min to create an inert environment using mass flow controller (D08-4D/ZM, Beijing Sevenstar, Huachuang Electronic Co., Ltd., Beijing, China). The nebulizer (Dr Hielscher UM20-1.6 MHz Sonic, Teltow, Germany) was used to generate an aerosol. The temperature was set to 775 °C at a ramp of 10 °C·min^−1^, gas flow rate to 400 mL/min and synthesis time to 45 min.

In order to investigate which catalytic system gave optimum yields, reaction vessel was charged with catalytic amounts (25, 50 and 100 mg/mL) of respective ferrocene and cobaltocene dissolved in toluene (Sigma-Aldrich Corporation, St Louis, MO, USA), which also served as the carbon source for carbon nanotube growth.

### 3.2. Design and Optimization of Formulations Using a Design Strategy

A three-factor Box-Behnken experimental design (BBD) was generated using Minitab^®^ V15 statistical software (Minitab^®^ Inc., State College, PA, USA) to determine the optimal pyrolysis conditions for the synthesis of vertically aligned MWCNTs. The variables employed in the experimental design were synthesis time, synthesis temperature and gas flow rate. The lower and upper limits for the synthesis time were 30 min and 60 min, respectively. The synthesis temperature limits were 650 °C and 950 °C and that of gas flow rates were 100 and 600 mL/min. The formulation variables were evaluated for their effect on the yield, length, external diameter and internal diameter of MWCNTs on both the quartz reactor tube and silicon wafer.

### 3.3. Functionalization of MWCNTs

The H_2_SO_4_:HNO_3_ functionalized MWCNTs were resuspended in SOCl_2_: DMF solution (20:1) and refluxed at 70 °C under N_2_ for 24 h (Scheme 1). The product was centrifuged at 5000 rpm for 20 min (Eppendorf Centrifuge, Eppendorf, Hamburg, Germany) and the precipitates washed with tetrahydrofuran (THF) at 5000 rpm for 20 min until the supernatant was crystal clear. The acylated carbon nanotubes (COCl-MWCNTs) were then dried in a vacuum oven at 70 °C for 24 h (Trade Raypa Espinar, Barcelona, Spain) [27].

DSPE PEG5000-4-arm (PEG-amine) was synthesised by reacting one equivalent DSPE PEG5000-amine (Nanocs Inc., New York, NY, USA) with 5 eq succinic anhydride in dichloromethane (Sigma Aldrich Corporation, St. Louis, MO, USA). The reaction was stirred at an ambient temperature for 24 h and evaporated to dryness. The product was reconstituted in deionised water and dialysed against water in a SnakeSkin dialysis tubing (Pierce-MWCO 3.5 kDa, Thermo Scientific, Waltham, MA, USA) for two days at a neutral pH. Dialysis tubing content was lyophilized in a Virtis Freeze Dryer (Gardiner, NY, USA) for 24 h. Then 1.5 eq dicyclohexylcarbodiimide (DCC) was mixed with 2 eq hydroxybenzotriazole (HOBt) in dichloromethane (DCM) purchased from Sigma-Aldrich Corporation (St. Louis, MO, USA). The mixture was added to the lyophilised DSPE PEG5000-COOH and reacted at room temperature for an hour. The 4 eq of 4-arm PEG-amine (Nanocs Inc., New York, NY, USA) was reacted with DSPE PEG5000-COOH in DCM for two days. The product was evaporated to dryness and reconstituted in deionised water and stirred for an hour at ambient temperature and filtered through 0.22 µm filter [28].

Acylated MWCNTs (0.2 mg/mL) were sonicated with 0.2 mM DSPE PEG5000-4-arm-(PEG-amine) for 1 h. The product was centrifuged at 240,000× *g* for 6 h (Beckman Coulter, Centrofriger-BL11, P-Selecta, Abrera, Spain) and supernatant was collected for characterization and drug release studies (Scheme 1).

### 3.4. Morphological and Molecular Spectroscopic Evaluation of the MWCNTs

MWCNTs were coated with palladium-gold using sputter coater (Emitech K550X, Emitech Ltd., Kent, England) for 4 min at 25 mA and 2 × 10^−1^ mbar and scanned in FEI Nova NanoLab 600 FEG-SEM/FIB (FEI Company, Hillsboro, OR, USA) to determine their morphology and size. SEM-EDX analysis was employed to confirm the elemental composition. TEM samples were dispersed in ethanol, ultrasonicated for 10 min (20 kHz sonicator, VibraCell, Sonics and Materials, Inc., Danbury, CT, USA), loaded on a copper grids, dried and loaded into FEI Tecnai T12 TEM operating at 120 kV for scanning and data processed using ImageJ software (National Institutes of Health, Bethesda, MD, USA).

In order to determine the G and D bands and the effect of functionalization, PEGylated and non-PEGylated MWCNTs were analysed using Raman Micro200 (Perkin Elmer, Beaconsfield, UK). Spectra were collected using laser beam of 785 nm wavelength with maximum output of 250 mW from 200 to 2500 cm^−1^ at 4 cm^−1^ resolution. KBr was mixed with MWCNTs, ground to fine particles, loaded on KBr die and pressed at 4-ton pressure under vacuum for 15 min with KBr press (ICL, International Crystal Labs, Garfield, NJ, USA) for molecular and vibrational transitions determination of MWCNTs. The pellet was scanned with Perkin Elmer Spectrum 2000 FTIR spectrometer (PerkinElmer Spectrum 100, Llantrisant, Wales, UK) at a resolution of 4 cm^−1^ with 10 accumulations with wavenumbers ranging from 4000 to 400 cm^−1^. MWCNTs were prepared for ^1^H NMR by dispersing 10 mg of Dex, PEG-MWCNTs and Dex-PEG-MWCNTs in 0.4 mL deuterated water, deuterated methanol and deuterated water, respectively. Samples were ultrasonicated for 2 min and analysed with 500 MHz Avance III spectrometer (Bruker Corporation, Billerica, MA, USA).

### 3.5. Thermogravimetric and Porositometric Analyses of MWCNTs

Thermal stability of MWCNTs were determined using a thermogravimetric analyzer (PerkinElmer TGA 4000, Llantrisant, Wales, UK). MWCNTs samples were heated from 30 °C to 950 °C under nitrogen gas purging environment at a constant flow rate of 20 mL/min and temperature ramp of 10 °C/min. Thermograms were generated as percentage weight loss vs. temperature and processed using PyrisTM software (PerkinElmer, Llantrisant, Wales, UK).

A porositometric analyzer (Micromeritics, ASAP 2020, Norcross, GA, USA) was employed to determine the porositometric properties of the pristine and functionalized MWCNTs. Sample was loaded in a sample tube, degassed and analysed by the Brunauer-Emmett-Teller (BET) N_2_ gas adsorption method at 77 K.

### 3.6. Particle Size, Zeta Potential, Crystallinity and Elemental Composition of MWCNTs

The 5 M HCl functionalized MWCNTs were suspended in deionized water at a concentration of 0.2 mg/mL, ultrasonicated for 2 h and measurements of particle size distributions and zeta potential were obtained using a ZetaSizer NanoZS (Malvern Instruments, Malvern, UK) instrument equipped with non-invasive backscatter technology set at a fixed angle of 173° and a light source with wavelength of 532 nm.

X-ray diffraction (XRD) patterns of the MWCNTs were examined on X-ray diffractometer (Philips, PANalytical X’pert PRO, X-ray diffraction system) at 40 kV and 40 mA with Cu Kα radiation (1.54060) equipped with K-beta filter. Each sample was scanned for 2 h because of high iron content in MWCNTs and the raw data processed with High Score Plus software (Malvern Panalytical Ltd., Malvern, UK).

### 3.7. Dex and Dex-PEG-MWCNTs Preparations and Drug Release Studies

Following 24 h reaction, Dex-MWCNTs were centrifuged at 5000 rpm for 10 min and dried in a vacuum oven for 24 h at room temperature. The centrifugation procedure was repeated thrice. Dex was also reacted with PEG-MWCNTs for 24 h, centrifuged and the supernatant collected for characterisation and drug release studies.

Dex-MWCNTs and Dex-PEG-MWCNTs were dispersed in 10 mL of 0.1 M phosphate buffered saline (PBS) and dialysis performed against 200 mL PBS buffer with pH 7.4, 6.5 and 5.5 respectively in an orbital shaker (YIHDER, Taiwan) at 37 °C with 30 rpm speed. Collection of 3 mL of the PBS buffer was done at various time intervals from 0 to 12 h. The sink conditions were maintained.

The λ_max_ of dexamethasone was determined using UV-VIS (Shimadzu Corporation, Kyoto, Japan) and was found to be 242 nm. Standard curve of Dex was then created using different concentrations and was used to determine concentrations in 3 mL aliquots of PBS buffer collected at various time intervals during a 12 h dialysis of the Dex-CNT and Dex-PEG-CNT constructs.

### 3.8. Cytotoxicity Evaluation of VA-MWCNTs

PC-12 cell lines were grown in RPMI1640 medium supplemented with 5% fetal bovine serum and 10% horse serum in an incubator at 37 °C and 5% CO_2_. A mixture of 1% penicillin and streptomycin solution was added to the culture medium to prevent bacterial contamination. In each well of 96 well plate, 6 × 10^4^ cells were added and incubated for 24 h with various concentrations (20, 200, 2000 and 20,000 µg/mL of each formulation (free Dex, equivalent Dex concentration coupled to PEGylated MWNT and sulfonitric acid treated MWCNTs. Controls (PC-12 cells not treated with any of the formulations-zero concentration group) for each formulation were also included in this study for comparison. A required amount of MTT (3-(4,5-Dimethylthiazol-2-yl)-2,5-Diphenyltetrazolium Bromide) was added to each well and further incubated for 4 h. Solubilization solution was added to solubilize formazan crystals and the absorbance read at 570 nm using Multilabel Reader (Victor X3, Perkin Elmer, Waltham, MA, USA).

## 4. Conclusions

Vertically aligned MWCNTs were successfully synthesised using the chemical vapor deposition technique following optimisation of the synthesis parameters such as time, temperature, location of substrate in the reactor, catalyst and gas flow rates. Carbon nanotubes were successfully purified and functionalized for dexamethasone loading. PEGylation enhanced the release of dexamethasone from the VA-MWCNTs. Functionalized carbon nanotubes had a very low cytotoxic effect in cells. Based on the above findings, functionalized VA-MWCNTs have a potential for sustained delivery of dexamethasone for an extended period to the diseased site. More work is under way for targeting ischemic sites using atrial natriuretic peptide antibody in stroke induced rats.

## Figures and Tables

**Table 1:** MWNT production using a combination of various catalysts.

Catalyst	Location	Mass before Run (g)	Mass after Run (g)	Final Mass (g)	Total Mass (g)	Yield %
Fe 25 mg/mL	Wafer	0.1114	0.1222	0.0108	0.1879	--
	QT	--	0.1771	0.1771		
Fe 25 mg/mLNi 1.25 mg/mL	Wafer	0.1278	0.1282	0.0004	0.3312	43.27
	QT	-	0.3312	0.3312		
Fe 25 mg/mLCo 1.25 mg/mL	Wafer	0.0933	0.0935	0.0002	0.5994	68.65
	QT	--	0.5992	0.5992		
Fe 25 mg/mLCo 1.25 mg/mLNi 1.25 mg/mL	Wafer	0.0435	0.0454	0.0019	0.2733	31.25
	QT	--	0.2714	0.2714		
Fe 50 mg/mLCo 2.5mg/mLNi 2.5 mg/mL	Wafer	0.1476	0.1481	0.0005	0.5097	63.14
	QT	--	0.5092	0.5092		

**Table 2:** Surface area of pristine and 5 M HCl functionalized MWCNTs.

Type	BET Surface Area (m^2^/g)	Pore Size (nm)	Pore Volume (m^3^/g)
Pristine MWCNTs	47.1563	13.08973	0.154316
5 M HCl-MWCNTs	144.0962	18.08957	0.651660
% Increase	67.27	27.64	76.32

## References

[1] Komane P.P., Choonara Y.E., Du Toit L.C., Kumar P., Kondiah P.P.D., Modi G., Pillay V. (2016). Diagnosis and treatment of neurological and ischemic disorders employing carbon nanotube technology. J. Nanomater..

[2] Iijima S. (1991). Helical microtubules of graphitic carbon. Nature.

[3] Lee G., Youk J.H., Lee J., Sul I.H., Yu W.R. (2016). Low-temperature grafting of carbon nanotubes on carbon fibers using a bimetallic floating catalyst. Diam. Relat. Mater..

[4] Lodhi N., Mehra N.K., Jain N.K. (2013). Development and characterization of dexamethasone mesylate anchored on multi walled carbon nanotubes. J. Drug Target..

[5] Roy S., David-Pur M., Hanein Y. (2017). Carbon nanotube growth inhibition in floating catalyst. Carbon.

[6] Tewari A., Sharma S.C. (2015). Effect of different carrier gases and their flow rates on the growth of carbon nanotubes. Phys. Plasmas.

[7] Rahman M., Younes H., Ni G., Zhang T., Al A. (2016). Synthesis and optical characterization of carbon nanotube arrays. Mater. Res. Bull..

[8] Psarros C., Lee R., Margaritis M., Antoniades C. (2012). Nanomedicine for the prevention, treatment and imaging of atherosclerosis. Nanomedicine.

[9] Veziri C.M., Pilatos G., Karanikolos G.N., Labropoulos A., Kordatos K. (2008). Growth and optimization of carbon nanotubes in activated carbon by catalytic chemical vapor deposition. Micropor. Mesopor. Mat..

[10] Allaedini G., Tasirini S., Aminayi P., Yaakob Z., Telib M. (2016). Carbon nanotubes via different catalysts and the important factors that affect their production: A review on catalyst preferences. Int. J. Nano Dimens..

[11] Ghaharpour F., Bahari A., Abbasi M., Akbar A. (2016). Parametric investigation of CNT deposition on cement by CVD process. Constr. Build. Mater..

[12] Voge C.M., Johns J., Raghavan M., Morris M.D., Stegemann J.P. (2013). Wrapping and dispersion of multiwalled carbon nanotubes improves electrical conductivity of protein-nanotube composite biomaterials. J. Biomed. Mater. Res. Part A.

[13] Khorrami S.A., Lotfi R. (2016). Influence of carrier gas flow rate on carbon nanotubes growth by TCVD with Cu catalyst. J. Saudi Chem. Soc..

[14] De Greef N., Zhang L., Magrez A., Forró L., Locquet J.P., Verpoest I., Seo J.W. (2015). Direct growth of carbon nanotubes on carbon fibers: Effect of the CVD parameters on the degradation of mechanical properties of carbon fibers. Diam. Relat. Mater..

[15] Acomb J.C., Wu C., Williams P.T. (2016). The use of different metal catalysts for the simultaneous production of carbon nanotubes and hydrogen from pyrolysis of plastic feedstocks. Appl. Catal. B Environ..

[16] Yuan Y., Lee T.R., Bracco G., Holst B. (2013). Contact Angle and Wetting Properties. Surface Science Techniques.

[17] Zhao Q., Bi J., Wang W., Sun G. (2016). A novel method of functionalizing carbon nanotubes via neutralization reaction. Mater. Lett..

[18] Eaton P., Quaresma P., Soares C., Neves C., de Almeida M.P., Pereira E., West P. (2017). A direct comparison of experimental methods to measure dimensions of synthetic nanoparticles. Ultramicroscopy.

[19] Razzazan A., Atyabi F., Kazemi B., Dinarvand R. (2016). In vivo drug delivery of gemcitabine with PEGylated single-walled carbon nanotubes. Mater. Sci. Eng. C.

[20] Besley N.A., Noble A. (2008). NMR chemical shifts of molecules encapsulated in single walled carbon nanotubes. J. Chem. Phys..

[21] Khorram R., Raissi H., Morsali A. (2017). Assessment of solvent effects on the interaction of Carmustine drug with the pristine and COOH-functionalized single-walled carbon nanotubes: A DFT perspective. J. Mol. Liq..

[22] Venditti I., Fontana L., Fratoddi I., Battocchio C., Cametti C., Sennato S., Mura F., Sciubba F., Delfini M., Vittoria M. (2014). Direct interaction of hydrophilic gold nanoparticles with dexamethasone drug: Loading and release study. J. Colloid Interface Sci..

[23] Roozbahani M., Kharaziha M., Emadi R. (2017). pH sensitive dexamethasone encapsulated laponite nanoplatelets: Release mechanism and cytotoxicity. Int. J. Pharm..

[24] Ketabi S., Rahmani L. (2017). Carbon nanotube as a carrier in drug delivery system for carnosine dipeptide: A computer simulation study. Mater. Sci. Eng. C.

[25] Zeinabad H.A., Zarrabian A., Saboury A.A., Alizadeh A.M.O., Falahati M. (2016). Interaction of single and multi wall carbon nanotubes with the biological systems: Tau protein and PC12 cells as targets. Sci. Rep..

[26] Gopinathan J., Quigley A.F., Bhattacharyya A., Padhye R., Kapsa R.M.I., Nayak R., Shanks R.A., Houshyar S. (2016). Preparation, characterisation, and in vitro evaluation of electrically conducting poly(ε-caprolactone)-based nanocomposite scaffolds using PC12 cells. J. Biomed. Mater. Res. Part A.

[27] Zare-Zardini H., Amiri A., Shanbedi M., Taheri-Kafrani A., Kazi S.N., Chew B.T., Razmjou A. (2015). In Vitro and in vivo study of hazardous effects of Ag nanoparticles and Arginine-treated multi walled carbon nanotubes on blood cells: Application in hemodialysis membranes. J. Biomed. Mater. Res. Part A.

[28] Liu Z., Chen K., Davis C., Sherlock S., Cao Q., Chen X., Dai H. (2008). Drug delivery with carbon nanotubes for in vivo cancer treatment. Cancer Res..

